# Is Brown Adipose Tissue Visualization Reliable on ^99m^Tc-Methoxyisobutylisonitrile Diagnostic SPECT Scintigraphy?

**DOI:** 10.1097/MD.0000000000002498

**Published:** 2016-01-15

**Authors:** Mahdi Haghighatafshar, Farinaz Farhoudi

**Affiliations:** From the Nuclear Medicine and Molecular Imaging Research Center, Namazi Teaching Hospital, Shiraz University of Medical Sciences, Shiraz, Iran (MH, FF).

## Abstract

The ^99m^Tc-MIBI has been used with great value as a diagnostic technique in patients with primary hyperparathyroidism. False-positive scans may occur due to misinterpretation of the physiologic distribution of the ^99m^Tc-MIBI. Reviewing consecutive SPECT scans, we evaluated this possibility and assessed how frequently brown adipose tissue (BAT) is seen on ^99m^Tc-MIBI scintigraphy.

Here, we retrospectively reviewed scans of consecutive patients who were evaluated for parathyroid adenomas from March 2015 to June 2015, using dual-phase (early and delayed) planar imaging and SPECT. We identified 60 patients (48 female and 12 male; mean age, 52.25 ± 15.20 years; range, 22–86 years).

We detected the presence of ^99m^Tc-MIBI uptake in BAT in 20 of 60 patients (33.33%) in the neck. Although the patients with ^99m^Tc-MIBI uptake in BAT were younger (mean age, 48.85 ± 15.27 years, range, 26–73 years) than the patients with no ^99m^Tc-MIBI uptake (mean age, 53.95 ± 15.07 years, range, 22–86 years), this difference was not statistically significant (*P* = 0.224). The percentage of female patients with BAT detection was higher (17/48 patients; 37.5%) than that of the male population (3/12 patients; 25%), this difference was not also statistically significant (*P* = 0.85).

In patient population referred to ^99m^Tc MIBI scintigraphy of the parathyroid glands, uptake of ^99m^Tc-MIBI in BAT should not be misinterpreted with ^99m^Tc-MIBI-avid-tumors. Fused SPECT/CT images (not SPECT-only) are necessary to distinguish BAT from bone, muscle, thyroid, myocardium, parathyroids, and other structures in the neck and chest.

## INTRODUCTION

Thallium-201 (^201^Tl) scintigraphy was the first radionuclide diagnostic method widely used in the 1980s for hyperfunctioning parathyroid localization.^1^ For parathyroid localization ^99m^Tc scintigram which is taken up only by the thyroid was digitally subtracted from the ^201^Tl scintigram which is taken up by both the thyroid and parathyroid glands.^2^ In 1989, Coakley et al^3^ used ^99m^Tc-methoxyisobutylisonitrile (^99m^Tc-MIBI) for parathyroid scintigraphy. Thallium superseded by the novel radionuclide because of several advantages such as higher energy (that makes it ideal for gamma camera imaging), better dosimetry and higher sensitivity.^4,5^ The subtraction of thyroid images obtained with ^123^I or ^99m^Tc from those obtained with ^99m^Tc-MIBI is a variation of this method.^6,7^ Taillefer et al^8^ found that ^99m^Tc-MIBI washes out more rapidly from thyroid gland than from hyperfunctioning parathyroid glands, so they suggested the use of a single-isotope dual-phase (early and delayed) scintigraphic method. ^99m^Tc tetrofosmin also has been used for parathyroid imaging, but the experiential data for this technique are scarce.^1^ In some adults, brown adipose tissue (BAT) is metabolically active and can cause false-positive scans due to misinterpretation of the physiologic distribution of the ^99m^Tc MIBI. Previously, BAT has been detected by some nuclear imaging methods such as single-photon imaging using ^123^I-metaiodobenzylguanidine,^9,10^ and by PET using ^18^F-FDG^11,12^ or ^99m^Tc-tetrofosmin^13^ in humans. Assessment of the visualized body during a ^99m^Tc MIBI scintigraphy of the heart or parathyroid glands is essential to exclude incidental tumoral uptake, which may be ^99m^Tc-MIBI-avid. As we know, there has been some new evidence of^1,14,15^^99m^Tc-MIBI uptake into BAT in humans so, reviewing consecutive SPECT scans we evaluated this possibility and assessed how frequently BAT is seen on ^99m^Tc-MIBI scintigraphy.

## MATERIALS AND METHODS

### Patient Population

Here we retrospectively reviewed scans of consecutive patients who were evaluated for parathyroid adenomas from March 2015 to June 2015, using dual-phase (early and delayed) planar imaging and SPECT. We identified 60 patients (48 female and 12 male; mean age, 52.25 ± 15.20 years, range, 22–86 years). As the study was retrospective and also anonymous, ethical approval was not necessary.

### Imaging Protocol and Analysis

All patients were injected intravenously with 740 to 925 MBq of ^99m^Tc-MIBI. We used a commercially available sestamibi kit (AEOI, Tehran, Iran) and according to the manufacturer's instructions, it was labeled and quality controlled.

### Imaging

At 20, 120, and 180 min after injection, 10-min anterior planar images were acquired in a 128 × 128 matrix, with a 20% window centered around the 140-keV photopeak, using a low-energy, high-resolution parallel collimator. SPECT images of the neck and chest were obtained using a rotating, calibrated General Electric Infinia Hawkeye 4 scintillation dual-head gamma camera(Fairfield , Connecticut, United States) equipped with a low energy high resolution parallel hole collimator immediately after planar imaging (at approximately 20 min).

## RESULTS

**FIGURE 1:**
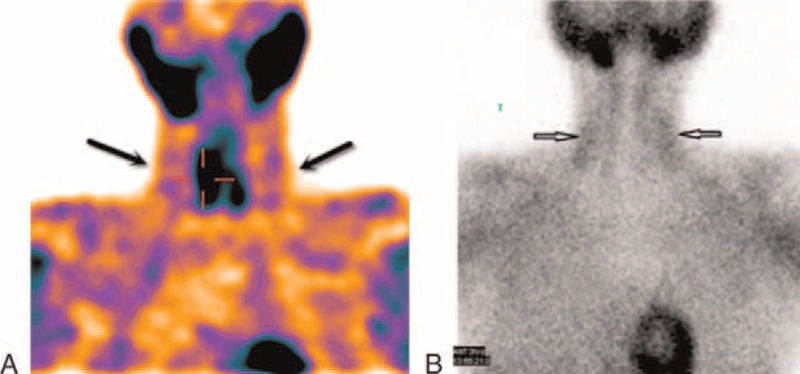
Early coronal slices of SPECT (A) and maximum-intensity projection (B) of a patient with primary hyperparathyroidism.^99m^Tc-MIBI-avid BAT is seen in the neck. ^99m^Tc-MIBI = ^99m^Tc-methoxyisobutylisonitrile.

**FIGURE 2:**
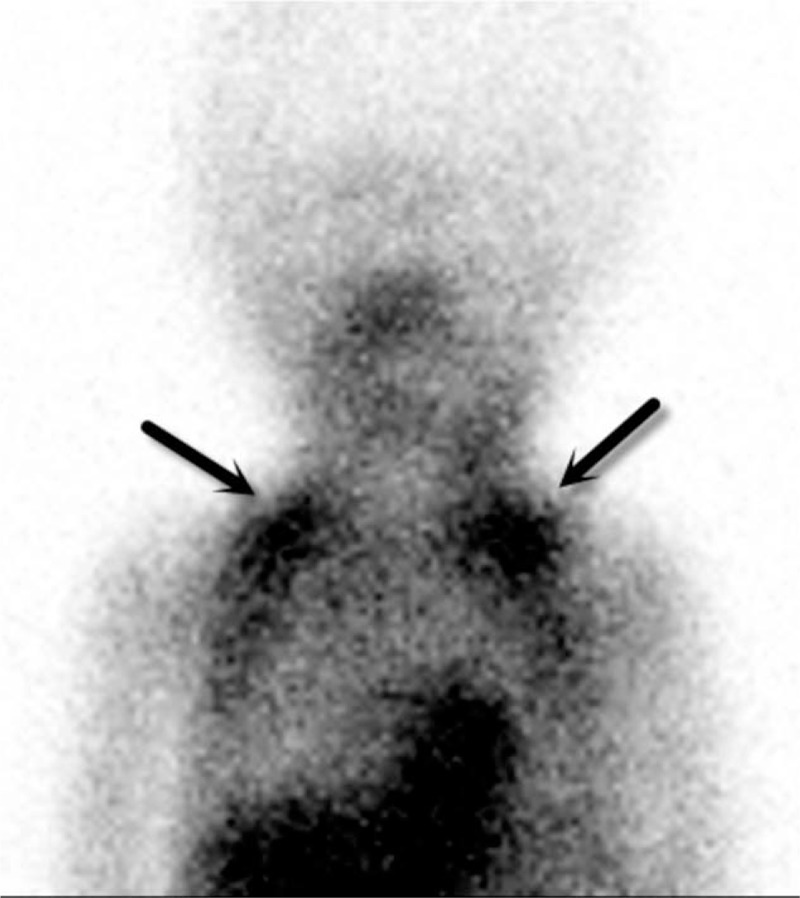
Scans performed on a 1-y-old infant with primary hyperparathyroidism with supraclavicular BAT (not included in data analysis).

We detected the presence of ^99m^Tc-MIBI uptake in BAT in 20 of 60 patients (33.33%) in the neck (Figures 1 and 2). When BAT was visualized, it was detected on both early and delayed scans. Although the patients with ^99m^Tc-MIBI uptake in BAT were younger (mean age, 48.85 ± 15.27 years, range, 26–73 years) than the patients with no ^99m^Tc-MIBI uptake (mean age, 53.95 ± 15.07 years, range, 22–86 years), this difference was not statistically significant (*P* = 0.224). The percentage of female patients with BAT detection was higher (17/48 patients; 37.5%) than that of the male population (3/12 patients; 25%), this difference was not also statistically significant (*P* = 0.85).

## DISCUSSION

After intravenous injection of ^99m^Tc-MIBI, this cationic radiotracer, which is also lipophilic, is distributed by blood flow in the body, cross the cellular membrane by passive transport, and become concentrated in the mitochondria.^16^ As hyperplastic parathyroid glands and parathyroid adenomas contain oxyphil cells laden with mitochondria, they can be visualized in ^99m^Tc-MIBI scintigraphy.^1^ Concentration of radiotracer is generally noticed in the infusion administered vein and it should not be mistaken with abnormal findings.^1^ Pioneer ^99m^Tc-MIBI usage as a radiotracer was limited to myocardial scintigraphy but became expanded due to considerable concentration in different type of tumors. Although normal parathyroid glands have no uptake, normal concentration of ^99m^Tc-MIBI is visualized in many tissues such as the thyroid gland, parotid and submandibular salivary glands, heart, and liver. Occasionally mild generalized uptake in the bone marrow and thymic uptake in young patients is visualized. Secondary to radiotracer secretion from the salivary glands, consideration of oral cavity accumulation in nonsevere degree is probable.^1^^99m^Tc-MIBI uptake also may be seen in BAT, metabolically active tissue, and should not be misinterpreted with disease, such as abnormal uptake in tumors, thyroid cancer, lymph nodes, parathyroid adenomas, or muscle.^17^ Despite reports of ^99m^Tc-MIBI uptake in BAT in rats,^18,19^ only a few data exist in the literature demonstrating ^99m^Tc-MIBI uptake in BAT in a human, using SPECT (or SPECT/CT).^14,15^ BAT is depicted by abundant vasculature and high mitochondrial metabolic activity and plays an important role in thermoregulation. Although additional studies are needed to understand the precise mechanism of ^99m^Tc-MIBI uptake in BAT but it is likely related to relatively high regional blood flow, possibly even more than to increased mitochondrial density and function.^14,18–20^ The frequency of BAT visualization in our study (SPECT-only) is very high in comparison with the frequency reported in studies with SPECT-CT in adults (33.33% for SPECT vs 6.3% for SPECT-CT). A higher frequency of BAT visualization with SPECT in this study might be related to the fact that no CT was obtained to ascertain that the tracer accumulation definitely is in BAT. In accordance with previous study^21^ that showed the importance of fused single-photon emission computed tomography (SPECT)/computed tomography (CT) images in radionuclide scintigraphy, BAT also could be clearly distinguished from bone, muscle, myocardium, thyroid, parathyroids, and other structures in the neck and chest (CT image of the expected region had to show fat tissue density and SPECT-only images of the patients had to show BAT in the same region).

## CONCLUSION

In patient population referred to ^99m^Tc MIBI scintigraphy of the parathyroid glands, uptake of ^99m^Tc-MIBI in BAT should not be misinterpreted with ^99m^Tc-MIBI-avid-tumors. Fused SPECT/CT images (not SPECT-only) are necessary to distinguish BAT from bone, muscle, thyroid, myocardium, parathyroids, and other structures in the neck and chest.

## References

[1] Eslamy HK Ziessman HA Parathyroid scintigraphy in patients with primary hyperparathyroidism: ^99m^Tc Sestamibi SPECT and SPECT/CT 1. *Radiographics* 2008; 28:1461–1476. 18794320 10.1148/rg.285075055

[2] Ferlin G Borsato N Camerani M New perspectives in localizing enlarged parathyroids by technetium-thallium subtraction scan. *J Nucl Med* 1983; 24:438–441. 6842292

[3] Coakley A Kettle A Wells C 99Tcm sestamibi—a new agent for parathyroid imaging. *Nucl Med Commun* 1989; 10:791–794. 2532313 10.1097/00006231-198911000-00003

[4] Bergenfelz A Tennvall J Valdermarsson S Sestamibi versus thallium subtraction scintigraphy in parathyroid localization: a prospective comparative study in patients with predominantly mild primary hyperparathyroidism. *Surgery* 1997; 121:601–605. 9186458 10.1016/s0039-6060(97)90046-5

[5] Fallahi B Haghighatafshar M Farhoudi F Comparative evaluation of the diagnostic accuracy of ^99m^Tc-sestamibi gated SPECT using five different sets of image acquisitions at stress and rest phases for the diagnosis of coronary artery disease. *Am J Nucl Med Mol Imaging* 2014; 4:10–16. 24380041 PMC3867725

[6] Casas AT Burke GJ Mansberger AR Prospective comparison of technetium-^99m^-sestamibi/iodine-123 radionuclide scan versus high-resolution ultrasonography for the preoperative localization of abnormal parathyroid glands in patients with previously unoperated primary hyperparathyroidism. *Am J Surg* 1993; 166:369–373. 8214295 10.1016/s0002-9610(05)80335-6

[7] Weber CJ Vansant J Alazraki N Value of technetium 99m sestamibi iodine 123 imaging in reoperative parathyroid surgery. *Surgery* 1993; 114:1011–1018. 8256204

[8] Taillefer R Boucher Y Potvin C Detection and localization of parathyroid adenomas in patients with hyperparathyroidism using a single radionuclide imaging procedure with technetium-99m-sestamibi (double-phase study). *J Nucl Med* 1992; 33:1801–1807. 1328564

[9] Fukuchi K Tatsumi M Ishida Y Radionuclide imaging metabolic activity of brown adipose tissue in a patient with pheochromocytoma. *Exp Clin Endocrinol Diab* 2004; 112:601–603. 10.1055/s-2004-830407 15578336

[10] Okuyama C Sakane N Yoshida T ^123^I-or ^125^I-metaiodobenzylguanidine visualization of brown adipose tissue. *J Nucl Med* 2002; 43:1234–1240. 12215564

[11] Hany TF Gharehpapagh E Kamel EM Brown adipose tissue: a factor to consider in symmetrical tracer uptake in the neck and upper chest region. *Eur J Nucl Med Mol Imaging* 2002; 29:1393–1398. 12271425 10.1007/s00259-002-0902-6

[12] Cohade C Osman M Pannu HK Uptake in supraclavicular area fat (“USA-Fat”): description on ^18^F-FDG PET/CT. *J Nucl Med* 2003; 44:170–176. 12571205

[13] Fukuchi K Ono Y Nakahata Y Visualization of interscapular brown adipose tissue using ^99m^Tc-tetrofosmin in pediatric patients. *J Nucl Med* 2003; 44:1582–1585. 14530470

[14] Higuchi T Kinuya S Taki J Brown adipose tissue: Evaluation with 201Tl and 99mTc-sestamibi dual-tracer SPECT. *Ann Nucl Med* 2004; 18:547–549. 15515758 10.1007/BF02984575

[15] Belhocine T Shastry A Driedger A Detection of 99mTc-sestamibi uptake in brown adipose tissue with SPECT-CT. *Eur J Nucl Med Mol Imaging* 2007; 34:149. 17021807 10.1007/s00259-006-0244-x

[16] Arbab AS Koizumi K Toyama K Uptake of technetium-99m-tetrofosmin, technetium-99m-MIBI and thallium-201 in tumor cell lines. *Cancer Treat Rep* 1983; 67:238. 8790217

[17] Goetze S Lavely WC Ziessman HA Visualization of brown adipose tissue with 99mTc-methoxyisobutylisonitrile on SPECT/CT. *J Nucl Med* 2008; 49:752–756. 18413387 10.2967/jnumed.107.048074

[18] Baba S Engles JM Huso DL Comparison of uptake of multiple clinical radiotracers into brown adipose tissue under cold-stimulated and nonstimulated conditions. *J Nucl Med* 2007; 48:1715–1723. 17873137 10.2967/jnumed.107.041715

[19] Kyparos D Arsos G Georga S Assessment of brown adipose tissue activity in rats by 99mTc-sestamibi uptake. *Physiol Res* 2006; 55:653–659. 16497107 10.33549/physiolres.930890

[20] Justo R Oliver J Gianotti M Brown adipose tissue mitochondrial subpopulations show different morphological and thermogenic characteristics. *Mitochondrion* 2005; 5:45–53. 16075478 10.1016/j.mito.2004.09.003

[21] Haghighatafshar M Gheisari F Ghaedian T Importance of heparin provocation and SPECT/CT in detecting obscure gastrointestinal bleeding on 99mTc-RBC scintigraphy: a case report. *Medicine* 2015; 94:e1325. 26313771 10.1097/MD.0000000000001325 PMC4602913

